# A comprehensive survey of error measures for evaluating binary decision making in data science

**DOI:** 10.1002/widm.1303

**Published:** 2019-02-08

**Authors:** Frank Emmert‐Streib, Salisou Moutari, Matthias Dehmer

**Affiliations:** ^1^ Predictive Society and Data Analytics Lab, Faculty of Information Technology and Communication Sciences Tampere University Tampere Finland; ^2^ Mathematical Sciences Research Centre, School of Mathematics and Physics Queen's University Belfast Belfast UK; ^3^ Institute for Intelligent Production, Faculty for Management University of Applied Sciences Upper Austria Steyr Campus Austria; ^4^ Department for Biomedical Computer Science and Mechatronics UMIT—The Health and Life sciences University Hall in Tyrol Austria; ^5^ College of Computer and Control Engineering Nankai University Tianjin P. R. China

**Keywords:** classification, data science, decision making, error measures, machine learning, statistics

## Abstract

Binary decision making is a topic of great interest for many fields, including biomedical science, economics, management, politics, medicine, natural science and social science, and much effort has been spent for developing novel computational methods to address problems arising in the aforementioned fields. However, in order to evaluate the effectiveness of any prediction method for binary decision making, the choice of the most appropriate error measures is of paramount importance. Due to the variety of error measures available, the evaluation process of binary decision making can be a complex task. The main objective of this study is to provide a comprehensive survey of error measures for evaluating the outcome of binary decision making applicable to many data‐driven fields.

This article is categorized under:

Fundamental Concepts of Data and Knowledge > Key Design Issues in Data Mining
Technologies > Prediction
Algorithmic Development > Statistics

## 1 INTRODUCTION

An important part of any scientific field is to make decisions based on available information. In its most prevalent form, this decision making is binary, that is, one selects between two options. Examples of such problems include the decision of a medical doctor for or against the surgery of a patient, the decision of a stock broker to buy or not to buy a security or the decision of an investor to provide venture capital or not to a company.

In this study, we provide a comprehensive survey of error measures for evaluating the outcome of binary decision making problems. These measures can be applied to any problem that can be cast as a classification problem and are therefore of great interest for many fields, including biomedical science, economics, information science, management, politics, medicine, natural science, social science, and web science. We will show that there is a variety of error measures available, making the evaluation process of the outcome of binary decision making a complex task.

Despite the importance of the problem, there are surprisingly few review or survey articles devoted to a comprehensive overview of this topic. Examples for such contributions can be found in Ferri, Hernández‐Orallo, and Modroiu (2009), Fielding and Bell (1997), Han, Pei, and Kamber (2011), Parker (2013) and Sokolova and Lapalme (2009). Importantly, their focus is different in various aspects including the accessibility and completeness compared to our study. For instance, in Sokolova and Lapalme (2009) only little discussion on the error measures is presented and important measures like the false discovery rate or the normalized mutual information are omitted. There are also review articles with a particular domain of application in mind, see e.g., Baldi, Brunak, Chauvin, Andersen, & Nielsen, 2000, focusing on the secondary structure of proteins and the alignment of amino acid sequences. Unfortunately, such presentations make them difficult to follow for scientists having an interest outside such a particular application domain because the presented discussions use a problem‐specific jargon that is cumbersome to convert into generic formulations. As a consequence, the benefit of such presentations is frequently tightly bound to narrow application fields. Furthermore, there are also advanced reviews available (Powers, 2011), that aim at the expert level providing many technical details.

For all of these reasons, in our survey paper, we are aiming at an accessible level of description for general purpose applications relevant for any data‐driven field. This is especially important in the emerging era of data science (Emmert‐Streib, Moutari, & Dehmer, 2016; Hardin et al., 2015) because this multidisciplinary field does not aim at a single application domain only but provides systematic approaches for any data generating field.

## 2 UNDERLYING METHODS OF DECISION MAKING

Before we start with surveying the error measures, we want to set the context by briefly discussing the methodology used for binary decision making.

The most common method used for binary decision making is a two‐class classifier. Prominent examples for such methods are support vector machines, decision trees, random forests, linear discriminant analysis, and neural networks (Breiman, 2001; Clarke, Fokoue, & Zhang, 2009; Schölkopf & Smola, 2002). Another method that makes binary decisions is a statistical hypothesis test (Lehman, 2005). In this case, one class corresponds to the “rejection of the null hypothesis” and the other class to the “acceptance of the null hypothesis.” A third type of method is more implicit or derived corresponding, for example, to a survival analysis comparing two patient groups (Kleinbaum & Klein, 2005). Such methods can be considered derived because, as in the case of the survival analysis, the decision is again made based on statistical hypothesis testing. For comparing survival curves one can use, for example, the Log‐rank or Mantel–Haenszel test.

It is interesting to note that the following error measures can not only be applied to the results of computational methods but also experimental ones. For instance, a patient being sick is consulting a medical doctor and a diagnostic test is conducted. The test is performed experimentally by, for example, analyzing a blood sample in a laboratory, but the result is binary indicating for instance the presence or absence of a disease. Other examples can be found in sociology where survey questions may be assessed in a similar way.

Overall, this means there are a large variety of different methods, computational and experimental ones, that can be used for obtaining binary decisions. However, regardless of the nature of the utilized method the following error measures can be applied for the evaluation of the outcome.

## 3 SUMMARIZING THE OUTCOME OF DECISION MAKING

For our following considerations we need a common ground that connects all the different methods discussed in the previous section. This can be achieved by utilizing a contingency table also called a confusion matrix.

A contingency table allows a convenient summarization of binary decision making. In the following, we assume we have two classes called +1 and −1. Here the indicators of the two classes are labels or nominal numbers (also called categorical numbers). The outcome of the decision making can be one of the following four cases:

1. The actual outcome is class +1 and we predict a +1.
2. The actual outcome is class +1 and we predict a −1.
3. The actual outcome is class −1 and we predict a +1.
4. The actual outcome is class −1 and we predict a −1.

It is convenient to give these four cases four different names. We call them as:

1. True positive: TP
2. False negative: FN
3. False positive: FP
4. True negative: TN

**Figure 1:**
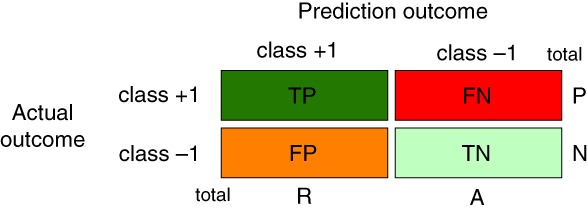
A summary of a binary decision making in the form of a contingency table

The purpose of a contingency table is to summarize these results, as shown in Figure 1.

It is easy to see that these four quantities characterize the outcome of a binary decision making completely. For this reason we are calling them the four fundamental error measures. All error measures we will discuss in the following sections will be based on these four fundamental errors. In order to facilitate the understanding, we will utilize the contingency table whenever beneficial for explaining different error measures.

We would like to note that we use the term “fundamental error measures” to emphasize the importance of these four measures from all other error measures discussed later. Mathematically, we will see that all following measures are functions of the four fundamental error measures. Hence, for these measures the TP, FP, TN and FN are *independent variables*.

## 4 OVERVIEW OF ERROR MEASURES

**Figure 2:**
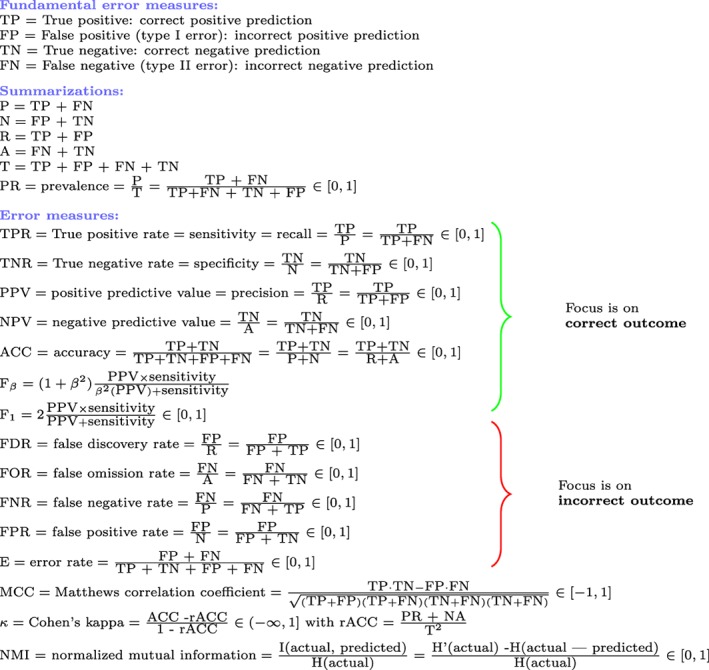
Overview of different error measures for binary decision making

In Figure 2, we show an overview of many different error measures that can be used for evaluating binary decision making. There are three broad groups of errors. The first group focuses on correct outcome, the second on incorrect outcome and the third on both. In the following section we will discuss each of these measures.

### 4.1 True positive rate and true negative rate

The true positive rate (TPR) and true negative rate (TNR) are defined by:

(1) $$\mathrm{TPR}=\text{sensitivity}=\frac{\mathrm{TP}}{P}=\frac{\mathrm{TP}}{\mathrm{TP}+\mathrm{FN}}\in \left[0,1\right],$$

(2) $$\mathrm{TNR}=\text{specificity}=\frac{\mathrm{TN}}{N}=\frac{\mathrm{TN}}{\mathrm{TN}+\mathrm{FP}}\in \left[0,1\right].$$

The definitions ensure that both measures are bound between 0 and 1. For an error‐free classification we obtain FN = FP = 0 which implies TPR = TNP = 1. On the other hand, for TP = TN = 0 we obtain TPR = TNP = 0. In the literature, the TPR is also called sensitivity and the TNR is called specificity (Flach, 2012).

**Figure 3:**
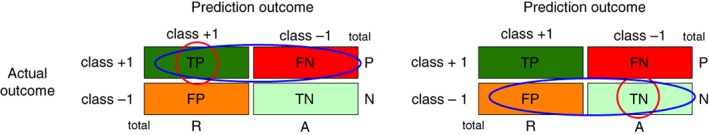
Left: true positive rate. Right: true negative rate

It is important to note that both quantities utilize only half of the information contained in the confusion matrix. The TPR uses only values from the first row and the TNR only values from the second row. In Figure 3 we highlight this by encircling the used fundamental errors. For simplicity, we refer to the first row as P‐level and the second row as N‐level. Hence, the TPR uses only values from the P‐level and the TNR values from the N‐level.

From Figure 3, it is clear that both measures are symmetric with respect to the utilized information; that is, just the roles of the classes are exchanged. This formulation enables to remember the measures more easily.

### 4.2 Positive predictive value and negative predictive value

The positive predictive value (PPV) and negative predictive value (NPV) are defined as:

(3) $$\mathrm{PPV}=\text{precision}=\frac{\mathrm{TP}}{R}=\frac{\mathrm{TP}}{\mathrm{TP}+\mathrm{FP}}\in \left[0,1\right],$$

(4) $$\mathrm{NPV}=\frac{\mathrm{TN}}{A}=\frac{\mathrm{TN}}{\mathrm{TN}+\mathrm{FN}}\in \left[0,1\right].$$

The definitions ensure that both measures are bound between 0 and 1. For an error‐free classification, we obtain FN = FP = 0 which implies PPV = NPV = 1. On the other hand, for TP = TN = 0 we obtain PPV = NPV = 0. In the literature, the PPV is also called precision (Fawcett, 2006).

**Figure 4:**
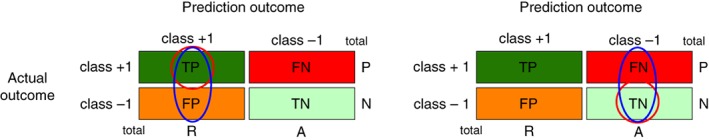
Left: positive predictive value. Right: negative predictive value

It is important to note that like TPR and TRN, the quantities PPV and NPV are estimated using only half of the information contained in the confusion matrix. The PPV uses only values from the first column and the NPV only values from the second column. In Figure 4 we highlight this by encircling the used fundamental errors. For simplicity, we refer to the first column as R‐level and the second column as A‐level. Hence, the PPV uses only values from the R‐level and the NPV values from the A‐level.

From Figure 3, it can be observed that both measures are symmetric with respect to the utilized information just the roles of the classes are exchanged. Such a formulation enables to remember the measures more easily.

### 4.3 Accuracy

The accuracy (A) is defined as:

(5) $$A=\frac{\mathrm{TP}+\mathrm{TN}}{\mathrm{TP}+\mathrm{TN}+\mathrm{FP}+\mathrm{FN}}=\frac{\mathrm{TP}+\mathrm{TN}}{P+N}=\frac{\mathrm{TP}+\mathrm{TN}}{R+A}\in \left[0,1\right].$$

The above definition ensures that the accuracy is bound between 0 and 1. For an error‐free classification, we obtain FN = FP = 0 which implies A = 1. On the other hand, for TP = TN = 0 we obtain A = 0. Another terminology used to refer to the accuracy, in the context of clustering evaluation, is the Rand index. In contrast with the quantities TPR, TNR, PPV and NPV, the accuracy uses all values in the confusion matrix.

### 4.4 *F*‐score

The general definition of the *F*‐score is:

(6) $$F_{\beta }=\left(1+\beta^{2}\right)\frac{\mathrm{PPV}\times \text{sensitivity}}{\beta^{2}\left(\mathrm{PPV}\right)+\text{sensitivity}}.$$

**Figure 5:**
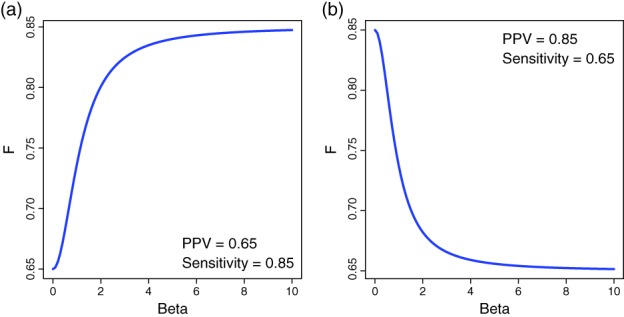
Behavior of the *F*‐score in dependence on the parameter *β*. The used values for PPV and sensitivity are mentioned in the figures

In this equation, the parameter *β* can assume values in the interval [0, ∞]. The parameter *β* enables to change the weighting between the PPV and the sensitivity. In Figure 5, we show an example for two different value pairs of PPV and sensitivity. It can be observed that for *β* = 0, the *F*‐score corresponds to the PPV whereas for *β* → ∞ it corresponds to the sensitivity. Intermediate values of *β* enable to obtain “averaged” *F*‐score values.

For *β* = 1, one obtains the *F*_1_‐score as:

(7) $$F_{1}=2\frac{\mathrm{PPV}\times \text{sensitivity}}{\mathrm{PPV}+\text{sensitivity}}\in \left[0,1\right].$$

The *F*_1_‐score is the harmonic mean of PPV and sensitivity, where the harmonic mean is defined as:

(8) $$\frac{1}{F_{1}}=\frac{1}{n}\left(\frac{1}{\mathrm{PPV}}+\frac{1}{\text{sensitivity}}\right).$$

The *F*_1_‐score uses three of the four fundamental errors, namely TP, FP and FN.

### 4.5 False discovery rate and false omission rate

The false discovery rate (FDR) and false omission rate (FOR) are defined as:

(9) $$\mathrm{FDR}=\frac{\mathrm{FP}}{R}=\frac{\mathrm{FP}}{\mathrm{FP}+\mathrm{TP}}\in \left[0,1\right],$$

(10) $$\text{FOR}=\frac{\mathrm{FN}}{A}=\frac{\mathrm{FN}}{\mathrm{FN}+\mathrm{TN}}\in \left[0,1\right].$$

The above definitions ensure that both measures are bound between 0 and 1. For an error‐free classification, we obtain FN = FP = 0, which implies FDR = FOR = 0. On the other hand, for TP = TN = 0, we obtain FDR = FOR = 1.

**Figure 6:**
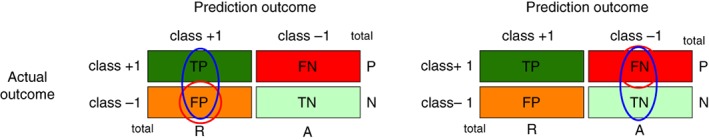
Left: false discovery rate. Right: false omission rate

Both FDR and FOR quantities utilize only half of the information contained in the confusion matrix. The FDR uses only values from the first column and the FOR only values from the second column. In Figure 6, we highlight this by encircling the used fundamental errors. That means the FDR uses information from the R‐level but in contrast to the PPV, which uses also information from this level, it focuses on failure by forming the quotient of FP and R. Similarly, the FOR uses only information from the A‐level, forming the quotient of FN and A.

From Figure 6 it is clear that both measures are symmetric with respect to the utilized information and just the roles of the classes are exchanged.

Frequent application domains for the FDR and the FOR are biology, medicine, and genetics (Farcomeni, 2008; Genovese & Wasserman, 2006).

### 4.6 False negative rate and false positive rate

The false negative rate (FNR) and false positive rate (FPR) are defined as:

(11) $$\mathrm{FNR}=\frac{\mathrm{FN}}{P}=\frac{\mathrm{FN}}{\mathrm{FN}+\mathrm{TP}}\in \left[0,1\right],$$

(12) $$\mathrm{FPR}=\frac{\mathrm{FP}}{N}=\frac{\mathrm{FP}}{\mathrm{FP}+\mathrm{TN}}\in \left[0,1\right].$$

From their definitions, both measures are bound between 0 and 1. For an error‐free classification, we have FN = FP = 0, which implies FNR = FPR = 0. On the other hand, for TP = TN = 0 we obtain FNR = FPR = 1.

**Figure 7:**
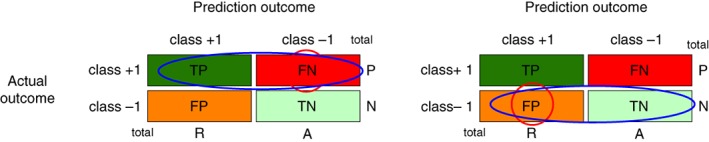
Left: false negative rate. Right: false positive rate

Furthermore, it is important to note that both quantities use only half of the information contained in the confusion matrix. The FNR uses only values from the first row whereas the FPR uses only values from the second row. In Figure 7, we highlight this by encircling the used fundamental errors.

From Figure 7, it can be again observed that both measures are symmetric with respect to the utilized information just the roles of the classes are exchanged.

### 4.7 Matthews correlation coefficient

A common issue when applying machine learning (ML) techniques to a real‐world problem is dealing with an imbalanced target variable. When applying ML methods to unbalanced data, the Matthews correlation coefficient (MCC) turned out to be useful (Matthews, 1975). It has been used to evaluate the performance of typical two‐class classification problems (Baldi et al., 2000). The MCC metric has been introduced first by Matthews to assess the performance of protein secondary structure prediction (Matthews, 1975):

(13) $$\mathrm{MCC}=\frac{\mathrm{TP}\cdot \mathrm{TN}-\mathrm{FP}\cdot \mathrm{FN}}{\sqrt{\left(\mathrm{TP}+\mathrm{FP}\right)\left(\mathrm{TP}+\mathrm{FN}\right)\left(\mathrm{TN}+\mathrm{FN}\right)\left(\mathrm{TN}+\mathrm{FN}\right)}}\in \left[-1,1\right]$$

**Figure 8:**
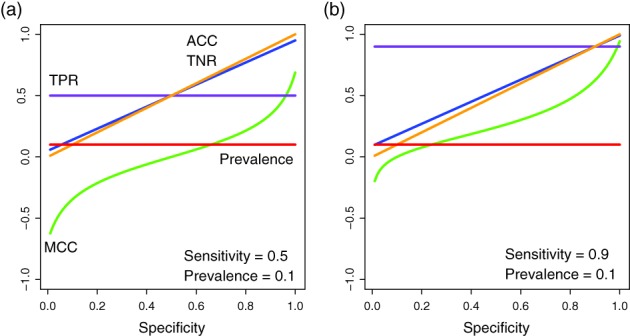
Behavior of the MCC according to the specificity value and the relationship between MCC and ACC, TPR and TNR. The used values for prevalence and sensitivity are mentioned in the figures

MCC ranges from −1 to +1. A value of −1 indicates that the prediction is completely wrong; a value of +1 indicates perfect prediction. MCC = 0 means that we consider a random classification where the model predictions have no detectable correlation to the true results. In Figure 8, we present some numerical results on the behavior of the MCC. For the shown simulations we assumed a fixed prevalence of 0.1 and a fixed sensitivity of 0.5 (left side) and 0.9 (right). The values of the specificity have been varied from 0.0 to 1.0 and all fundamental errors have been derived from this, including the shown ACC, TPR, and TNR. The chosen value for the prevalence ensures a strong imbalance between both classes.

In Figure 8a, low specificity values correspond in general to poor classification results as indicated by very low ACC values and negative values of the MCC. With an increasing specificity, the TNR and ACC increase both with a similar slope. However, due to the imbalance of the classes and the way we defined our model keeping the TPR constant, the increasing values of ACC are misleading. Indeed, the MCC increases but not linearly and much slower than the ACC. Moreover, the highest achievable value for MCC is only 0.68 in contrast to 0.95 for ACC. In Figure 8b, the sensitivity is much higher and, hence, the difference between ACC and MCC becomes reduced but is still clearly noticeable.

Due to the fact that an imbalance in the categories is a frequent problem encountered in analyzing data, the MCC is used throughout all application domains of data science.

### 4.8 Cohen's kappa

The next measure we will discuss is called Cohen's kappa, *κ* (Cohen, 1960). It has been essentially used as a measure to assess how well the classifier performed as compared to how well it would have performed simply by chance. That means a model has a high kappa value if there is a big difference between the accuracy and the null error rate. Formally expressed, Cohen's kappa has been defined by Cohen (1960) as:

(14) $$\kappa =\frac{\mathrm{ACC}-\text{rACC}}{1-\text{rACC}}\in \left(-\infty ,1\right].$$

Here, ACC is the accuracy and rACC denotes the randomized accuracy defined as:

(15) $$\text{rACC}=\frac{\mathrm{PR}+\mathrm{NA}}{T^{2}}.$$

Usually, Cohen's kappa has been used as a measure of agreement between two categorical variables in various ML applications across several disciplines, including epidemiology, medicine, psychology and sociology (see e.g., Saczynski et al., 2012; Umesh, Peterson, & Sauber, 1989).

### 4.9 Normalized mutual information

The normalized mutual information is an information‐theoretic measure, which has been used extensively in various applications (Principe, Xu, Zhao, & Fisher, 2000; Rost & Sander, 1993; Shridhar, Bartlett, & Seagrave, 1998). In most of these studies the normalized mutual information was applied to classification tasks and other learning problems.

When reviewing and assessing the evaluation criteria for learning problems, Baldi et al. (2000) defined the normalized mutual information as follows:

(16) $$\mathrm{NMI}=\frac{\mathrm{TP}}{T}\log\frac{\mathrm{TP}}{T}\frac{\mathrm{TN}}{T}\log\frac{\mathrm{TN}}{T}\frac{\mathrm{FP}}{T}\log\frac{\mathrm{FP}}{T}\frac{\mathrm{FN}}{T}\log\frac{\mathrm{FN}}{T},$$

(17) $$-\frac{\mathrm{TP}}{T}\log\left(\frac{\mathrm{TP}+\mathrm{FP}}{T}\frac{\mathrm{TP}+\mathrm{FN}}{T}\right)-\frac{\mathrm{FN}}{T}\log\left(\frac{\mathrm{TP}+\mathrm{FN}}{T}\frac{\mathrm{TN}+\mathrm{FN}}{T}\right),$$

(18) $$-\frac{\mathrm{FP}}{T}\log\left(\frac{\mathrm{TP}+\mathrm{FP}}{T}\frac{\mathrm{TN}+\mathrm{FP}}{T}\right)-\frac{\mathrm{TN}}{T}\log\left(\frac{\mathrm{TN}+\mathrm{FN}}{T}\frac{\mathrm{TN}+\mathrm{FP}}{T}\right).$$

In fact, this expression can be simplified (Baldi et al., 2000) to:

(19) $$\mathrm{NMI}=\frac{\mathrm{TP}}{T}\log\frac{\mathrm{TP}}{T}\frac{\mathrm{TN}}{T}\log\frac{\mathrm{TN}}{T}\frac{\mathrm{FP}}{T}\log\frac{\mathrm{FP}}{T}\frac{\mathrm{FN}}{T}\log\frac{\mathrm{FN}}{T},$$

(20) $$-\frac{\mathrm{TP}}{T}\log\left(\frac{R}{T}\frac{P}{T}\right)-\frac{\mathrm{FN}}{T}\log\left(\frac{P}{T}\frac{A}{T}\right),$$

(21) $$-\frac{\mathrm{FP}}{T}\log\left(\frac{R}{T}\frac{N}{T}\right)-\frac{\mathrm{TN}}{T}\log\left(\frac{A}{T}\frac{N}{T}\right).$$

Many variants of the normalized mutual information measure have been defined and applied (see e.g., Hu & Wang, 2008; Principe et al., 2000; Rost & Sander, 1993; Shridhar et al., 1998; Wallach, 2006). For instance, Hu and Wang (2008) define the normalized mutual information measures on the so‐called augmented confusion matrix. This matrix has been defined by adding one column for a rejected class onto a conventional confusion matrix (Hu & Wang, 2008). Wallach (2006) considers normalized confusion matrices representing models and formulates the normalized mutual information measures on that matrix as well as other error measures such as Precision and Recall. By comparing the defined models, Wallach (2006) draws the conclusion that classical measures such as Precision and Recall may be misleading when evaluating classifier using only these measures.

### 4.10 Area under receiver operator characteristic curve

The final error measure we are presenting is the *area under receiver operator characteristic* (AUROC) curve (Bradley, 1997; Hanley & McNeil, 1982). In contrast to all previous measures discussed in this study, the AUROC curve can only be obtained via a construction process, rather than directly derived from the contingency table. The reason for this is that it does not make use of the optimal threshold of a classifier for deciding how to categorize data samples. Instead, this threshold can be derived, as we will show at the end of this section.

The first step of this construction process is to derive the ROC curve and the second is the integration of this curve to obtain the area under the curve. For constructing a ROC curve we need to obtain pairs of TPR and FPR (i.e., TPR_*i*_, FPR_*i*_). That means the ROC curve presents the TPR in dependence on the FPR. Due to the fact that the TPR is also the sensitivity and the FPR is also 1‐specificity, an alternative representation would be the sensitivity in dependence on 1‐specificity.

Let us assume that we have a dataset with *n* samples. Regardless of the method used, we can obtain either a score *s*_*i*_ or a probability *p*_*i*_ for every sample as an indicator of the membership for class +1 (and analogously values for class −1). In the following, we will discuss the construction based on probabilities but the discussion is similar using scores. Based on these probabilities, a decision is obtained by thresholding these values. That means for

(22) $$p_{i}>p_{t},$$

we decide to place sample *i* into class +1 and otherwise in class −1. By rank ordering all *p*_*i*_ for all samples in increasing order we obtain:

(23) $$p\left[1\right]\leq p\left[2\right]\leq \ldots \leq p\left[n\right].$$

Now we apply successively all possible thresholds to obtain two groups, one group corresponding to class +1 and the other to class −1. This results in total in *n* + 1 different thresholds and, hence, groupings:

(24) $$|p\left[1\right]\leq p\left[2\right]\leq \ldots \leq p\left[n\right],$$

(25) $$p\left[1\right]\leq |\;p\left[2\right]\leq \ldots \leq p\left[n\right],$$

(26) ⋮

(27) $$p\left[1\right]\leq p\left[2\right]\leq \ldots \leq |\;p\left[n\right],$$

(28) $$p\left[1\right]\leq p\left[2\right]\leq \ldots \leq p\left[n\right]|.$$

For each of these groupings we can calculate the four fundamental errors and based on these, every error measure shown in Figure 2, including the TPR and FPR can also be calculated. Overall, this results in *n* + 1 pairs of (TPR_*i*_, FPR_*i*_) for *i* ∈ {1,  … , *n* + 1} from which the ROC curve is constructed.

**Figure 9:**
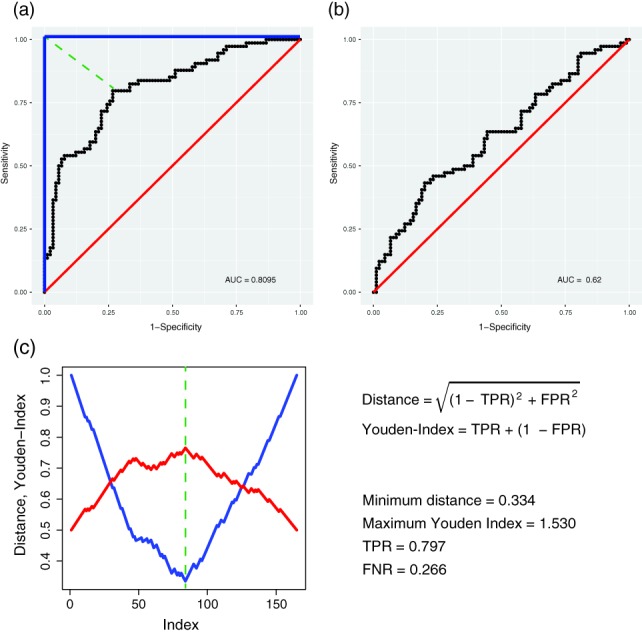
In the first row two examples for ROC curves are shown resulting from two logistic regression analyses. The second row shows results for Figure 9a for the distance to the ROC curve and the Youden‐Index leading to optimal thresholds

In Figure 9, we present some examples for ROC curves (in black) as a result from two logistic regression analyses. From Figure 9a,b, the smaller the AUROC value the closer is the ROC curve to the main diagonal (in red) corresponding to a random classification, which would result in AUROC = 0.5. On the other hand, the best possible classifier achieves AUROC = 1.0 which would correspond to a ROC curve shown in blue.

In addition to providing the AUROC values, ROC curves can also be used to determine the optimal threshold for the classifier itself since they were obtained without using this threshold, even if already determined in some way. However, in order to obtain such an optimal threshold we need to define an optimization function. In the literature, there are two frequent choices for this (Fawcett, 2006). The first is the distance from the ROC curve to the upper left corner, that is (TPR = 1, FPR = 0) given by:

(29) $${\text{distance}}_{i}=\sqrt{{\left(1-{\mathrm{TPR}}_{i}\right)}^{2}+{\mathrm{FPR}}_{i}^{2}}$$

and the second is called the Youden‐Index (Youden, 1950) given by:

(30) $$\text{Youden}‐{\text{Index}}_{i}={\mathrm{TPR}}_{i}+\left(1-{\mathrm{FPR}}_{i}\right).$$

The optimal threshold is then obtained by finding

(31) $$i_{\mathrm{opt}}^{d}=\underset{i}{\mathrm{argmin}}\left\{{\text{distance}}_{i}\right\},$$

(32) $$i_{\mathrm{opt}}^{Y}=\underset{i}{\mathrm{argmax}}\left\{\text{Youden}‐{\text{Index}}_{i}\right\}.$$

In general, these indices do not have to result in identical values, however, for the example shown in Figure 9a this is the case, as illustrated in Figure 9c. We would like to note that for reasons of visibility we scaled the displayed Youden‐Index between 0 and 1.

After obtaining the optimal threshold one can derive any error measure, including the values of the TPR and FPR. For the ROC curve in Figure 9a, this leads to TPR = 0.797 and FNP = 0.266.

## 5 EVALUATING OUTCOME AS MULTIVARIATE PROBLEM

Now we are arriving at a point where we need to ask ourselves which measure to use when studying binary decision making problems? The general answer to this question is that there is no single measure that would be appropriate in all possible situations under all feasible conditions but, instead, one needs to realize that evaluating binary decision making should be done in a multivariate way. This means that, usually, we need to use more than one error measure to evaluate the outcome in order to avoid false interpretations of the prediction results.

In order to illustrate this aspect, we present an example. For this example, we simulate the values in the contingency table according to a model. Specifically, this means that we are defining an error model. To simplify the situation, we define this error model in the proportions of the four fundamental errors TP, FP, TN and FP, we are calling pTP, pFP, pTN and pFP. Each of these proportions can assume values between 0 and 1, and the four quantities sum up to 1, that is:

(33) pTP+pFP+pTN+pFP=1.

Furthermore, if we multiply each of these proportions with *T* (the total number of samples) we recover the four fundamental errors, that is:

(34) TP=T⋅pTP,

(35) TN=T⋅pTN,

(36) FP=T⋅pFP,

(37) FN=T⋅pFN.

**Figure 10:**
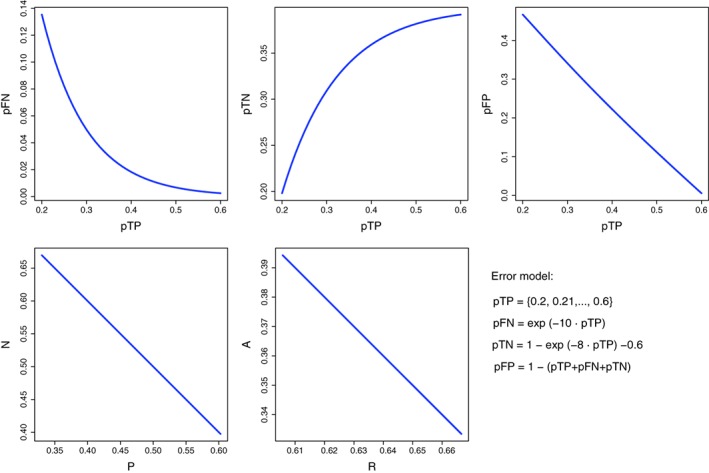
Visualization of the assumed error model that defines the proportions of the four fundamental errors

In Figure 10, we depict a visualization of the assumed error model that defines the proportions of the four fundamental errors. It is easy to check that the sum of these four quantities equals always 1, for all possible values shown. The simulations start by assuming that pTP takes its values in the interval from 0.2 to 0.6 in step sizes of 0.01. Based on this, the values of pFN and pTN are determined by some exponential functions, as indicated in Figure 10. Finally, the value of pFP ensures just the conservation of the total probability, hence, no functional relation needs to be defined for this proportion. Overall, the model starts with a poor decision outcome corresponding to low pTP and pTN value and improves toward higher values corresponding to a better decision making. Furthermore, the model is unbalanced in the sizes of the classes as can be seen from the values of P and N. It is also unbalanced in the predictions (see R and A).

**Figure 11:**
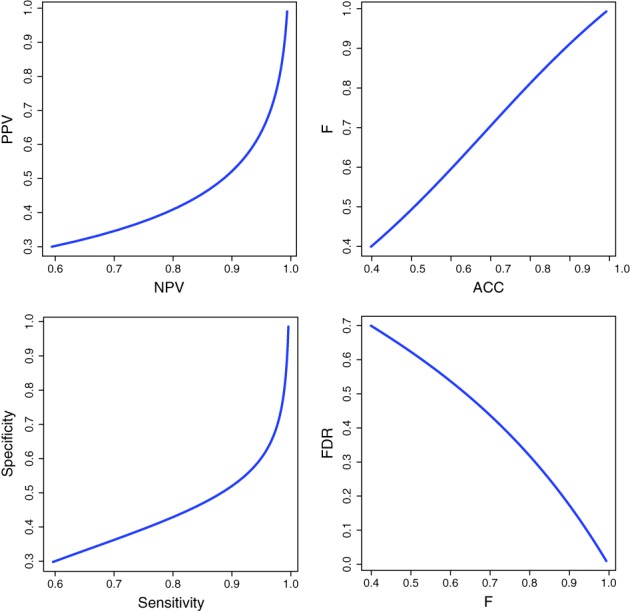
Dashboard view of seven error measures according to the error model shown in Figure 10

In Figure 11, we present results for seven error measures that are obtained using the values from our error model. All of these pairwise dashboards show nonlinearities, at least to some extend. The most linear (but not exactly linear) correspondence is exhibited between the ACC and the *F*‐score followed by the relationship between the FDR and the *F*‐score. For instance, for pTP = 0.2 we obtain ACC = *F* = 0.39 indicating low classification performance but taking in addition the value of FDR = 0.7 into consideration we see that 70*%* of all samples classified as class +1 are false. From this perspective, the classification results are even very poor. From the specificity versus sensitivity plot one can see that class +1 is much easier to recover than class −1 because there is a strong nonlinearity between these two error measures with a faster increase in the values of the sensitivity (TPR). This is reflected similarly in the PPV.

### 5.1 Comparing binary decision making methods

**Figure 12:**
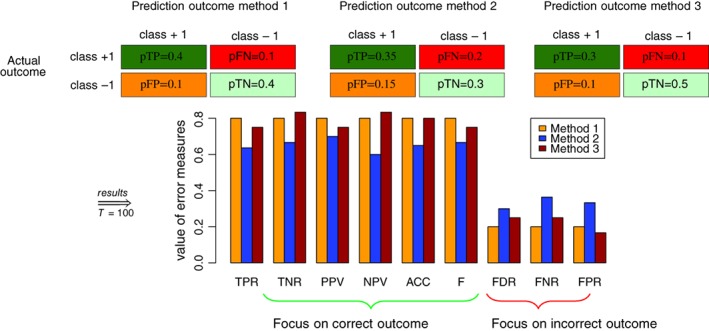
Outcome of three binary decision making methods. The error measures are evaluated for *T* = 100 samples

In the above examples we have shown that changes in the four fundamental errors (as defined by an error model) can lead to nonlinear effects in the dependent errors, for instance, in FDR. However, this type of issue is not the only problem in evaluating binary decision making. Another problem is given by evaluating two instead of one binary decision making methods. In order to demonstrate this type of problem we show in Figure 12 the outcome of three binary decision making methods.

Specifically, in the first part of Figure 12 we show the proportion of the four fundamental errors for the three methods. Here we assumed that the application of a method to a dataset results in the shown errors and all three methods are applied to the same dataset. In order to demonstrate the problem we consider two scenarios. The first scenario corresponds to a comparison of the outcome of Method 1 with Method 2 and the second scenario to a comparison of the outcome of Method 1 with Method 3.

For Scenario 1, from Figure 12 we see that

- pTP_1_ > pTP_2_
- pTN_1_ > pTN_2_
- pFN_1_ < pFN_2_
- pFP_1_ < pFP_2_

that means, the true predictions for Method 1 are always higher than for Method 2 and the false predictions for Method 1 are always lower than for Method 2. For this reason it seems obvious that Method 1 performs better than Method 2 regardless of what fundamental error measure is used and no matter if one considers just one of these or combinations. The result of the comparison is that Method 1 performs always better than Method 2.

However, for Scenario 2, shown in Figure 12 comparing Method 1 with Method 3, we see that

- pTP_1_ > pTP_3_
- pTN_1_ < pTN_3_
- pFN_1_ = pFN_3_
- pFP_1_ = pFP_3_

First, we observe that the false predictions are identical. Second, the proportion of true positives is higher for Method 1 but the proportion of true negatives is higher for Method 2. Third, the absolute value of the distance of the true predictions is equal for the positive and negative classes, that is:

(38) $$\mathrm{\Delta pTP}={\mathrm{pTP}}_{1}-{\mathrm{pTP}}_{3}=0.1$$

(39) $$\mathrm{\Delta pTN}={\mathrm{pTN}}_{1}-{\mathrm{pTN}}_{3}=-0.1$$

but the sign is different. Taking this information together one can see that without further information one cannot decide if Method 1 is better than Method 3 or vice versa.

This can be further clarified by looking at different error measures that are a function of the four fundamental errors. In Figure 12, we show results for *T* = 100. For Scenario 1, we see that all error measures that focus on positive outcome are higher for Method 1 (in orange) than for Method 2 (in blue) and all error measures that focus on negative outcome are lower for Method 1 than for Method 2. In contrast, for Scenario 2, we see that there are some error measures that focus on positive outcome are higher for Method 1 (in orange) than for Method 3 (in brown) and some are lower for Method 1 (in orange) than for Method 3. For instance,

(40) $${\mathrm{TPR}}_{1}>{\mathrm{TPR}}_{3},$$

(41) $${\mathrm{TNR}}_{1}<{\mathrm{TNR}}_{3}.$$

Similar results hold for error measures that focus on negative outcome. For instance,

(42) $${\mathrm{FDR}}_{1}<{\mathrm{FDR}}_{3},$$

(43) $${\mathrm{FPR}}_{1}>{\mathrm{FPR}}_{3}.$$

Hence, the outcome of the comparison to decide which method is better depends on the used error measure. This example demonstrates the need to argue in favor of one or several error measures because this will influence the result of the comparison. However, in order to select one or several measures we need to add additional information beyond what is available in Figure 12 and this additional information needs to be domain‐specific. However, domain‐specific information results in domain‐specific problems, for instance, in biology, medicine, finance or social sciences.

## 6 OCCURRENCE OF ERROR MEASURES

**Figure 13:**
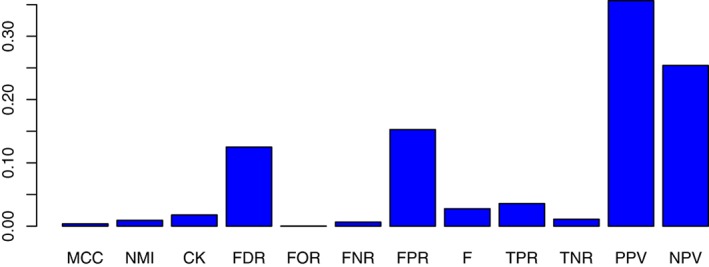
Comparison of error measures with respect to their relative usage frequency. The frequency numbers have been obtained from Google Scholar

Finally, we want to take a look into the literature to see what error measures are frequently used. In Figure 13, we show a comparison of the relative usage frequency of error measures. The underlying frequency numbers on which these relative frequencies are based on have been obtained from Google Scholar. For instance, for the PPV we find 644,000 different articles in which this term appears and for all terms combined we find 1,807,481 articles. This gives a relative usage frequency of the PPV of 35.6%. Overall, the PPV, NPV, FDR and FPR are dominating the literature.

From Figure 13, one can clearly see that not all error measures are used equally frequently but that there are tremendous differences in their usage. In order to obtain information about the application areas we repeat a similar analysis but now we evaluate the relative frequencies for application fields. For obtaining this information we use Scopus because Google Scholar does not provide information about the application fields.

**Figure 14:**
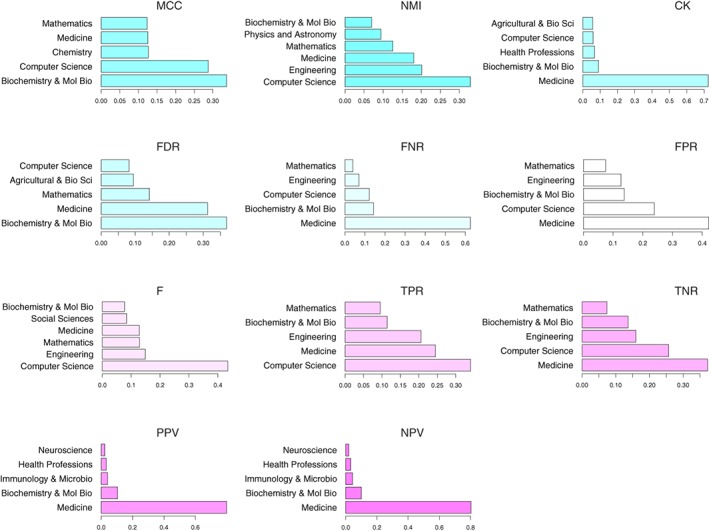
Application field specific to relative usage frequency of error measures. The frequency numbers have been obtained from Scopus

In Figure 14, we show the results from this analysis for 11 error measures. For each error measure, we show the top five application fields. From these results it is also obvious that the application fields are not equally frequent but error measure specific. For instance, the FNR is most frequently used in medicine and to a much lesser extend in computer science. In contrast, for the *F*‐score the roles are reversed. One can also see that medicine is for six out of nine error measures the field with the most frequent applications. Furthermore, medicine is among the top five application fields for all 11 error measures. A possible reason for this is that in medicine the conducted analyses need to be stringent due to the fact that patients may be harmed in case of erroneous analyses. For this reason, one cannot afford to neglect (patient) information by focusing on only a few error measures. Another reason is that patient‐related analyses usually require approval by senior scientists. All these factors underline the need to consider as much information as possible captured by error measures. Furthermore, it also indicates that there is no simple answer to the question which error measure should be used because each error measure provides a different perspective about an underlying problem.

## 7 CONCLUSION

In this study, we provided a comprehensive overview of many error measures that can be used for evaluating binary decision making in data science (Emmert‐Streib et al., 2016). We were aiming at an accessible level of description and avoided application domain‐specific formulations in order to make knowledge transfer easier for general purpose applications in fields such as biomedical science, economics, management, politics, medicine, natural science, psychology, or social science.

In summary, there are many error measures but none is adequate in isolation to capture effectively all different outcome scenarios of binary decision making. For this reason, in practical applications, combinations of these error measures need to be selected in order to obtain a dashboard overview of the complex relationships which allow realistic interpretations. Furthermore, we have seen that the contingency table provides a very useful summary of the outcome from decision making on which the calculation of many error measures is based on, with the exception of the AUROC.

Finally, we would like to note that, in practice, resampling methods (Efron, 1981; Molinaro, Simon, & Pfeiffer, 2005; Schumacher, Holländer, & Sauerbrei, 1997), for example, cross validation, will be applied for estimating variabilities and to protect against overfitting. This will allow estimating the mean value of error measures as well as the corresponding standard error. Hence, error measures are only one side of the medal when evaluating the outcome of binary decision making. Resampling methods complement these error measures by enabling estimates of the distribution of error measures.

## CONFLICT OF INTEREST

The authors have declared no conflicts of interest for this article.
